# Possible mechanisms of spermatogenic dysfunction induced by viral infections: Insights from COVID‐19

**DOI:** 10.1002/rmb2.12625

**Published:** 2025-01-22

**Authors:** Keisuke Okada, Chanhyon Kin, Yosuke Yamashita, Shun Kawamura, Katsuya Sato, Koji Chiba, Hideaki Miyake

**Affiliations:** ^1^ Department of Urology Kobe City Medical Center West Hospital Kobe Japan; ^2^ Division of Urology, Department of Organs Therapeutics Kobe University Graduate School of Medicine Kobe Japan

**Keywords:** covid‐19, male infertility, SARS‐COV‐2, spermatogenesis

## Abstract

**Background:**

As the COVID‐19 pandemic nears resolution in 2024, the mechanisms by which SARS‐CoV‐2 and other viral infections induce spermatogenic dysfunction remain poorly understood. This review examines the mechanisms by which viral infections, particularly COVID‐19, disrupt spermatogenesis and highlights the implications for male reproductive health. While reports suggest that spermatogenic dysfunction caused by COVID‐19 is mild and transient, these findings may have broader applications in understanding and treating spermatogenic dysfunction caused by future viral infections.

**Methods:**

The PubMed database was searched to identify original and review articles investigating the mechanisms by which viral infections, particularly SARS‐CoV‐2, contribute to spermatogenic dysfunction.

**Main Findings:**

SARS‐CoV‐2 affects the testis through multiple mechanisms, including ACE2 receptor‐mediated entry, direct viral damage, inflammatory response, blood–testis barrier disruption, hormonal imbalance, oxidative stress, and impaired spermatogenesis. The combination of these factors can disrupt testicular function and highlights the complexity of the effects of COVID‐19 on male reproductive health.

**Conclusion:**

COVID‐19 may disrupt spermatogenesis through direct testicular infection, systemic inflammation, hormonal disruption, and oxidative stress. Ongoing research, vaccination efforts, and clinical vigilance are essential to address these challenges and develop effective treatment and prevention strategies.

## Graphical abstract

This review examines the current understanding of COVID‐19's impact on male reproductive health, particularly on testicular function. The study outlines multiple mechanisms through which SARS‐CoV‐2 can affect the testes, including direct viral damage, inflammatory responses, hormonal imbalances, and oxidative stress. The authors emphasize the complexity of these effects and stress the importance of continued research, vaccination efforts, and clinical vigilance to address the potential long‐term consequences of COVID‐19 on male fertility.

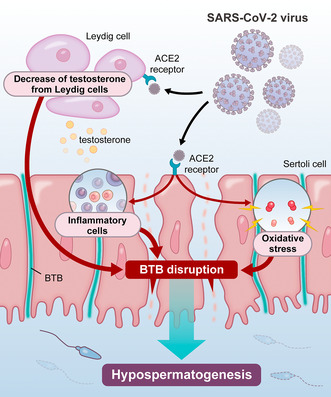

## 1 INTRODUCTION

Coronaviruses are the largest family of positive‐stranded RNA viruses, which currently includes 30 members.^1^, ^2^ They are widely distributed in nature and are capable of infecting humans and other mammals.^3^, ^4^ In 2019, a highly contagious virus emerged from Wuhan, China, known as SARS‐CoV‐2.^5^, ^6^ The 2019 novel coronavirus disease (COVID‐19) caused by SARS‐CoV‐2 elicits common clinical manifestations, such as fever, dry cough, and in severe cases, multiple organ damage.^7^, ^8^, ^9^ Although the acute phase of the pandemic has subsided as of 2024 in many regions because of widespread vaccination, immunity, and improved treatments, COVID‐19 remains an endemic disease with periodic outbreaks and ongoing concerns, particularly with respect to new variants.^10^, ^11^, ^12^, ^13^

SARS‐CoV‐2 has significantly impacted global health, with over 500 million confirmed cases and millions of deaths occurring worldwide.^14^ Its high transmission rate and illness severity have resulted in unprecedented public health measures, including lockdowns, travel restrictions, and widespread vaccination campaigns.^15^ The development of effective vaccines and treatments has been an important step in reducing the severity and spread of this disease.^16^ Despite these efforts, however, SARS‐CoV‐2 continues to pose challenges because of its ability to mutate and evade immune responses.^17^ This has resulted in the emergence of new variants that can potentially reduce vaccine efficacy and lead to breakthrough infections.^18^

In addition to its well‐documented respiratory effects, SARS‐CoV‐2 can affect multiple organ systems, including the male reproductive tract.^19^ Studies have suggested that the testes may be particularly vulnerable to COVID‐19, raising concerns about the potential long‐term effects on male fertility and reproductive health.^19^, ^20^ The involvement of the male reproductive system is significant as the testes play a major role in hormone production and spermatogenesis, which are vital to male fertility. The virus causes orchitis, a condition characterized by inflammation of the testes, which leads to the potential disruption of these important functions.

During SARS‐CoV‐2 transmission, the receptor angiotensin I‐converting enzyme 2 (ACE2) is required for virus cell entry,^21^, ^22^ and transmembrane serine protease 2 (TMPRSS2) is necessary for priming the S protein.^19^ Both proteins are co‐expressed in testicular cells, such as Leydig and Sertoli cells.^8^, ^9^, ^10^ This suggests that the virus targets the testis during infection. ACE2 is the receptor that facilitates the entry of the virus into host cells. Its expression in the testes indicates a potential route for the virus to infect and damage testicular tissue.^23^ TMPRSS2 further contributes to this process by priming the viral spike protein to enhance its ability to fuse with host cell membranes. Male patients account for the majority (56%–73%) of COVID‐19 infections^4^, ^24^, ^25^; however, the potential effects of the virus on the male reproductive system remain unclear.

The blood–testis barrier, while normally protective, may inadvertently trap the virus within the seminiferous tubules, prolonging its presence and potential effects.^26^ The blood–testis barrier (BTB) is a physiological barrier that separates the developing sperm cells from the bloodstream, thus preventing the entry of harmful substances and immune cells; however, this barrier may also sequester the virus within the testes, allowing it to persist and cause prolonged damage. This may lead to chronic inflammation and immune responses that further harm testicular tissue and impair function.

Significant alterations in semen parameters, hormone levels, and testicular histology occur in COVID‐19 patients.^27^, ^28^, ^29^ This has prompted studies to identify the underlying mechanisms through which SARS‐CoV‐2 affects testicular function, which may include direct viral damage, immune‐mediated inflammation, oxidative stress, and disruption of the hypothalamic–pituitary–gonadal axis.^30^, ^31^, ^32^ Direct viral damage involves the virus infecting and replicating within testicular cells, which results in cell death and tissue damage.^33^, ^34^ Immune‐mediated inflammation occurs when the host's immune response to the virus causes collateral damage to the testes.^35^, ^36^, ^37^ Oxidative stress results from an imbalance between the production of reactive oxygen species and the body's ability to neutralize them, which results in cellular damage.^38^, ^39^, ^40^ The hypothalamic–pituitary–gonadal axis is a complex system that regulates reproductive function.^41^, ^42^, ^43^ Its disruption by the virus results in hormonal imbalances that affect fertility.^28^, ^44^, ^45^, ^46^

There are some reports suggesting that COVID‐19 impacts semen parameters, including reductions in sperm count, motility, and overall quality. However, some studies indicate these changes might be transient, as observed in the findings of Majzoub et al.,^47^ where partial recovery was noted over time.

Li et al.^48^ performed a study on semen quality in men recovering from COVID‐19 and found a significant reduction in sperm concentration, total sperm count, and motility. The study included 38 patients, with 23 having achieved clinical recovery and 15 in the acute stage of infection. Six patients (15.8%) had SARS‐CoV‐2 in their semen, including 4 of 15 (26.7%) at the acute stage and 2 of 23 (8.7%) in the recovery phase. There was no significant difference in semen quality based on age, urogenital disease history, days since onset, hospitalization, or clinical recovery. They hypothesized that the reduction in semen quality may result from the direct viral invasion of testicular tissue or a systemic inflammatory response.

Cakir et al.^49^ conducted a study comparing semen parameters in two groups: patients who underwent semen analysis before and after COVID‐19 diagnosis (*n* = 114) and an age‐matched control group without COVID‐19 (*n* = 114). The results showed significant reductions in semen volume, total sperm count, progressive motility, total motility, and normal morphology after SARS‐CoV‐2 infection. Fever during infection was identified as a key factor negatively affecting sperm concentration, while the duration since diagnosis (short‐term vs. long‐term) did not influence these changes. The study concludes that SARS‐CoV‐2 infection is associated with a decline in semen quality, potentially impacting male fertility, with some effects persisting long‐term.

Despite these reports, the long‐term prognosis remains uncertain. Considering that similar mechanisms may underlie spermatogenic dysfunction caused by future viral infections, this review aims to explore the potential mechanisms by which COVID‐19 could lead to sustained impairments in spermatogenesis.

In this review, we summarize the current understanding of the possible mechanisms by which viral infections, including SARS‐CoV‐2, induce spermatogenic dysfunction. We discuss evidence from both clinical studies and animal models, focusing on the molecular and cellular processes involved in testicular damage and how these viral infections may disrupt spermatogenesis. Specifically, we highlight mechanisms such as direct viral invasion of testicular cells, inflammatory responses, and oxidative stress, which have been observed in the context of COVID‐19. Furthermore, we explore the potential long‐term implications for male reproductive health and fertility. This review underscores the critical need for ongoing research to fully elucidate the impact of COVID‐19 and other viral infections on male fertility, to address concerns about possible long‐term decreases in fertility, and to inform strategies for managing and mitigating these effects. This is essential for ensuring appropriate care for affected individuals.

## 2 METHODS

We conducted a comprehensive literature review to explore the mechanisms of SARS‐CoV‐2‐induced spermatogenic dysfunction. PubMed was used as the primary database to retrieve relevant articles. We used search terms such as “SARS‐CoV‐2,” “COVID‐19,” “spermatogenesis,” “male infertility,” and “testicular dysfunction,” focusing on studies published from 2020 to the present. Only peer‐reviewed articles published in English were included to ensure reliability and accessibility.

After the initial search, we screened titles and abstracts to identify studies that specifically examined the effects of SARS‐CoV‐2 on male reproductive health, including direct viral effects on testicular tissue, inflammatory responses, and hormonal disturbances. Selected articles were fully reviewed and pertinent data on molecular mechanisms, clinical findings, and observed outcomes related to spermatogenesis were extracted and synthesized. Studies discussing similar mechanisms in other viral infections were also considered for comparative insights.

This approach allowed us to collect and evaluate a range of evidence that provides a comprehensive overview of how SARS‐CoV‐2 may affect male reproductive health and spermatogenesis.

## 3 BASIC PATHOLOGY OF SARS‐COV‐2 INFECTION

COVID‐19 is caused by the SARS‐CoV‐2 virus and primarily affects the respiratory system; however, it can also affect multiple organ systems. Pathogenesis begins with viral entry via the ACE2 receptor, expressed in the respiratory epithelium and also in testicular tissue.^50^ The SARS‐CoV‐2 virus replicates within host cells, resulting in cell damage and triggering an immune response.

The hallmark of severe COVID‐19 is a “cytokine storm,” which is an excessive inflammatory response leading to ARDS and multi‐organ failure.^51^ This hyperinflammation is characterized by increased levels of pro‐inflammatory cytokines, including IL‐6, TNF‐α, and IL‐1β.^52^ Pathological findings in COVID‐19 patients typically include diffuse alveolar damage, hyaline membrane formation, and inflammatory cell infiltration.^53^ Microthrombi formation in the pulmonary vasculature contributes to severe hypoxemia.^54^ COVID‐19 also affects the cardiovascular system, causing myocardial injury and arrhythmias. Viral infection is associated with neurological manifestations, ranging from anosmia to encephalopathy.^55^, ^56^ Coagulation abnormalities are frequently observed in severe cases, with elevated D‐dimer levels and a prothrombotic state.^57^ This can result in both venous and arterial thromboembolism, which further complicates the clinical picture.^58^

Endothelial cell dysfunction plays an important role in the pathogenesis of COVID‐19, contributing to both the inflammatory response and the prothrombotic state.^59^ The virus can directly infect endothelial cells, resulting in endotheliitis and microvascular dysfunction.^60^ Postmortem studies have revealed widespread organ involvement, including damage to the heart, kidneys, and liver, particularly in patients without preexisting conditions.^61^ This multi‐organ involvement underscores the systemic nature of severe COVID‐19.

The immune response to SARS‐CoV‐2 is complex and involves both innate and adaptive immunity. While a robust immune response is necessary for viral clearance, a dysregulated response can result in immunopathology.^62^ T‐cell exhaustion and lymphopenia are common features in severe cases.^63^

Understanding the pathology of COVID‐19 has resulted in the development of various therapeutics, including antivirals, immunomodulators, and anticoagulants^64^; however, the heterogeneity of the disease and the potential for long‐term sequelae highlight the need for continued studies.^65^

With respect to testicular involvement, studies have shown that SARS‐CoV‐2 affects male fertility. The testes express ACE2 receptors, rendering them susceptible to viral entry.^54^ Although direct viral entry into testicular tissue remains controversial, the inflammatory response and oxidative stress associated with COVID‐19 may disrupt normal testicular function.^55^

## 4 DIRECT VIRAL DAMAGE TO TESTES

The testis, which is required for male reproduction, is a potential target for SARS‐CoV‐2 infection. This susceptibility is primarily attributed to the presence of ACE2 receptors in testicular cells. ACE2 serves as the primary entry point for SARS‐CoV‐2 into host cells, which renders any ACE2‐expressing tissues vulnerable to infection.

Wang et al. examined the expression of ACE2 in adult human testes using single‐cell RNA‐sequencing data and found that ACE2 was primarily expressed in spermatogonia, Leydig cells, and Sertoli cells. This suggests that the testis are a potential target for SARS‐CoV‐2 infection, raising concerns about the effect of the virus on testicular function and male fertility.^20^

Direct viral damage to the testis has been observed in histopathological studies of COVID‐19 patients. Ma et al. examined testicular biopsy specimens from 12 deceased COVID‐19 patients. They reported significant seminiferous tubular injury, reduced Leydig cells, and mild lymphocytic inflammation. Interestingly, they also detected SARS‐CoV‐2 viral particles in testicular tissue by transmission electron microscopy, which provides direct evidence of viral invasion.^66^

The mechanism of testicular damage in COVID‐19 appears to be multifaceted. In addition to direct viral invasion, the inflammatory response triggered by SARS‐CoV‐2 infection may contribute to testicular injury. Li et al.^67^ proposed that the cytokine storm associated with severe COVID‐19 may increase the permeability of the blood–testis barrier, allowing immune cells and inflammatory mediators to infiltrate the testis and cause damage.

The potential for SARS‐CoV‐2 to persist in the testis has raised concerns about the long‐term effects on male fertility. Achua et al. detected SARS‐CoV‐2 in the testis of asymptomatic COVID‐19 patients up to 3 months following the initial diagnosis. They observed significant impairment of spermatogenesis and evidence of viral invasion in spermatogenic, Sertoli, and Leydig cells.^68^

## 5 HORMONAL DISRUPTION

The COVID‐19 pandemic has sparked interest in its multifaceted effects on human health, including its effect on the endocrine system. Male reproductive health has become scrutinized as several studies suggest that COVID‐19 may significantly affect male hormone levels, particularly testosterone and luteinizing hormone (LH). This section reviews key findings from recent studies of how COVID‐19 influences male hormonal balance.

Ma et al.^69^ conducted a comprehensive study on hormonal changes in COVID‐19 patients, which revealed a marked increase in circulating LH levels and a reduced testosterone ratio. The study included 81 men diagnosed with COVID‐19, whose hormonal profiles were compared with those of 100 healthy controls. The results indicated that the average LH level in COVID‐19 patients was significantly higher compared with that in the control group. This hypergonadotropic state, characterized by elevated LH levels, suggests a compensatory mechanism in response to reduced testosterone production, which suggests Leydig cell dysfunction.

Koç et al.^70^ reported a reduction in circulating testosterone levels post‐COVID‐19 infection. Their study involved 21 men recovering from COVID‐19. None of the patients required hospitalization at any time through the course of COVID‐19. A significant decrease in semen volume, percentage of total motility, percentage of progressive motility, and normal sperm morphology was observed following COVID‐19 infection. There was also a significant decrease in the T level of the patients after the COVID‐19 diagnosis.

Cinislioglu et al.^71^ determined the relationship between disease severity and hormonal changes and found that a decline in testosterone levels was directly proportional to the severity of the disease. This study involved 358 male COVID‐19 patients, which were categorized into mild, moderate, and severe groups based on clinical presentation and treatment requirements. The measured serum total testosterone level of the COVID‐19 group was significantly lower compared with that of the control group. The severe group exhibited a significant reduction in testosterone levels, accompanied by higher LH concentrations. These findings highlight the direct impact of systemic inflammation and viral load on endocrine function.

Yang et al.^29^ determined the effect of COVID‐19 on male sex hormones and semen parameters. They found significantly decreased testosterone to luteinizing hormone (T/LH) ratios in COVID‐19 patients compared with healthy controls, which suggested impaired Leydig cell function. In addition, they observed a higher prevalence of oligozoospermia and increased seminal leukocytes in COVID‐19 patients, indicating potential damage to spermatogenesis.

## 6 INFLAMMATORY RESPONSE AND BTB DISRUPTION

The BTB is formed near the basement membrane by a variety of junctions, including tight junctions (TJs), basal ectoplasmic specializations, gap junctions, and desmosome‐like junctions between two adjacent SCs.^72^ The BTB is anatomically much more complex compared with that of other blood–tissue barriers, such as the blood–brain barrier. In addition, the BTB may be divided into three components: anatomical, physiological, and immunological barriers.^73^ The junctions that restrict the passage of molecules and cells into or out of the BTB form the anatomical barrier. The physiological barrier consists of transporters that regulate the passage of substances, thus creating a microenvironment for spermatogenesis. The immunological barrier limits access to systemic immunity and sequesters the majority of the auto‐antigenic germ cells. A functional BTB relies on the complex interaction between the three components.^74^

The systemic inflammatory response, or cytokine storm, induced by COVID‐19 contributes to endocrine dysfunction.^75^ Ahmed et al.^76^ discussed the role of cytokines, particularly IL‐6 and TNF‐α, in the pathophysiology of COVID‐19. They described how increased levels of these cytokines are associated with severe disease outcomes, including detrimental effects on various bodily functions, such as endocrine disruption. Inflammatory cytokines, such as interleukin‐6 (IL‐6), negatively affect testosterone synthesis. In COVID‐19 patients, IL‐6 and TNF‐α levels were significantly higher compared with healthy individuals, whereas testosterone and inhibin B levels were significantly lower. Cinislioglu et al.^71^ provided evidence of decreased testosterone and inhibin B levels in COVID‐19 patients, linking these changes to increased levels of pro‐inflammatory cytokines, such as IL‐6 and TNF‐α. In addition, TNF‐α showed a positive correlation with age and IL‐6, whereas inhibin B had a negative correlation with TNF‐α. This suggests that men with symptomatic COVID‐19 tend to exhibit low testosterone and inhibin B levels, along with increased IL‐6 and TNF‐α. This emphasizes the harmful effects of SARS‐CoV‐2 on testicular function and immune response, which is independent of age. Such disruption affects the blood–testis barrier (BTB) and decreases the expression of important junctional proteins, such as occludin, claudin, and connexin‐43, which are essential for maintaining BTB integrity.^77^, ^78^, ^79^

COVID‐19 disrupts testicular function through the induction of inflammatory cytokines, including IL‐6. SARS‐CoV‐2 interacts with angiotensin‐converting enzyme 2 (ACE2) receptors, which are expressed on testicular cells, such as Sertoli and Leydig cells. During COVID‐19 infection, downregulation of ACE2 in testicular cells results in an increase in pro‐inflammatory cytokines, including IL‐6, which contributes to testicular inflammation and dysfunction. This inflammatory response can disrupt spermatogenesis, resulting in reduced sperm count and motility.^80^

Elevated IL‐6 levels are commonly observed in COVID‐19 patients, particularly for those with severe disease. This increase in IL‐6 is a marker of the cytokine storm associated with severe COVID‐19 and indicates the heightened inflammation and immune response in various body tissues, including the testes.^81^

## 7 OXIDATIVE STRESSES

COVID‐19‐induced oxidative stress is a potential factor contributing to male reproductive health problems. Oxidative stress induced during viral‐mediated local and systemic inflammation is responsible for testicular damage that affects germ cell and endocrine cell function.^82^ A decrease in sperm count, motility, number of normal spermatozoa, and an increase in DNA fragmentation are common features during the course of viral infections, although they generally resolve once the infection is cleared.^83^

The effect of oxidative stress on male fertility has been well‐documented. Agarwal et al.^84^ showed that the excessive production of reactive oxygen species (ROS) results in sperm membrane lipid peroxidation, DNA damage, and apoptosis. These effects can significantly impair sperm function and fertility. The SARS‐CoV‐2 virus induces a state of increased oxidative stress, which may exacerbate the detrimental effects on male reproductive health.^85^

Persistent viral shedding observed in some COVID‐19 cases raises concerns regarding potential sexual transmission, even after the viral load has disappeared from the blood.^86^ This prolonged presence of the virus in the male reproductive tract may result in persistent oxidative stress and inflammation, potentially causing long‐term damage to testicular tissue and function.

Oxidative stress can damage testicular tissue and affect Sertoli and Leydig cell function. Sertoli cells play an important role in spermatogenesis by providing nutritional and structural support to developing germ cells, whereas Leydig cells are responsible for testosterone production.^87^ Damage to these cells because of oxidative stress can result in disruptions in spermatogenesis and hormonal imbalances, further compromising male fertility. Recent studies suggest that SARS‐CoV‐2 may directly infect testicular cells through the ACE2 receptor.^20^ This direct viral infection may exacerbate oxidative stress induced damage to testicular tissue and function.

Moghimi et al.^88^ evaluated testicular tissue from autopsies of COVID‐19‐positive and negative males. COVID‐19 infection significantly reduced seminiferous tubule length, interstitial tissue, and the number of testicular cells. Increased expression of ACE2, BAX, and Caspase 3, along with decreased BCL2 expression, was observed. Oxidative stress markers, such as ROS production, were increased, and GSH activity was suppressed in COVID‐19 cases. Immunohistochemistry and TUNEL assays showed increased ACE2 expression and apoptotic cells. These findings suggest that COVID‐19 disrupts spermatogenesis through oxidative stress and induces apoptosis, resulting in reduced testosterone levels and compromised spermatogenesis.

Sengupta et al.^89^ evaluated the impact of COVID‐19 on male reproductive health. Out of 553 articles, 25 met the inclusion criteria, revealing that while there is little evidence of viral RNA in semen, COVID‐19 can affect seminal parameters, induce orchitis, and cause hypogonadism. Severe cases are associated with significant testicular histological damage, which is likely the result of inflammation and oxidative stress. Clinical evaluation of male reproductive health, particularly in fertility patients, is recommended. Long‐term effects on male reproduction are unknown and warrant further study.

Based on these findings, implementing strategies to reduce oxidative stress and support testicular function may be beneficial for men recovering from COVID‐19. Antioxidant supplementation and lifestyle changes aimed at reducing oxidative stress will help to protect against virus‐induced damage to the male reproductive system.^90^

### 7.1 COVID‐19 vaccines and male fertility: Evidence supporting safety and benefits

The safety of COVID‐19 mRNA vaccines (Pfizer‐BioNTech and Moderna) with respect to male reproductive health has been demonstrated in several well‐designed studies.^91^, ^92^

Gonzalez et al.^91^ conducted a comprehensive analysis of semen parameters (sperm concentration, motility, morphology, and volume) before and after vaccination and found no clinically significant changes. Specifically, their study of 45 healthy men who received two doses of the Pfizer‐BioNTech vaccine showed no significant decline in sperm parameters during the 70‐day follow‐up period.

Some studies reported transient minor changes in sperm motility, but these changes remained within normal physiological ranges and did not affect overall fertility potential.^92^

In contrast, SARS‐CoV‐2 infection itself poses significant risks to testicular function.^19^ Li and colleagues^67^ demonstrated that the virus can induce testicular inflammation and impair spermatogenesis. This finding is particularly important because it highlights the protective role of vaccination.

A meta‐analysis by Yang et al.^29^ showed a significant deterioration of semen parameters in COVID‐19 patients, with more pronounced effects in severe cases. This further emphasizes the importance of vaccination as a preventive measure.

## 8 LIMITATIONS

Studies of COVID‐19 and testicular dysfunction have several limitations. First, there is considerable heterogeneity in study design. The lack of standardized protocols among clinical trials makes it difficult to compare results and draw consistent conclusions. Second, most studies have focused on the short‐term effects of COVID‐19 on male fertility, with limited long‐term follow‐up. Thus, longer follow‐up is needed to fully understand the recovery process and potential long‐term effects on male fertility. Third, the effect of confounding factors, such as fever, medications, and comorbidities, are not always adequately considered. These factors can have a significant impact on testicular function but are often overlooked in trials. There is also a lack of direct viral detection in the testes. Although immunohistochemistry has confirmed the presence of IgG deposition, the absence of viral genomic components in testicular tissue or seminal plasma makes it difficult to confirm a direct effect of COVID‐19 on the testes. This is in contrast with other viruses, such as mumps, in which the direct invasion of the testis has been documented.^93^, ^94^, ^95^, ^96^ Finally, the mechanisms underlying testicular dysfunction in COVID‐19 are complex, involving a combination of inflammation, oxidative stress, and potential autoimmune responses. This complexity makes it difficult to isolate the specific contributing factors.

## 9 CONCLUSIONS

**FIGURE 1:**
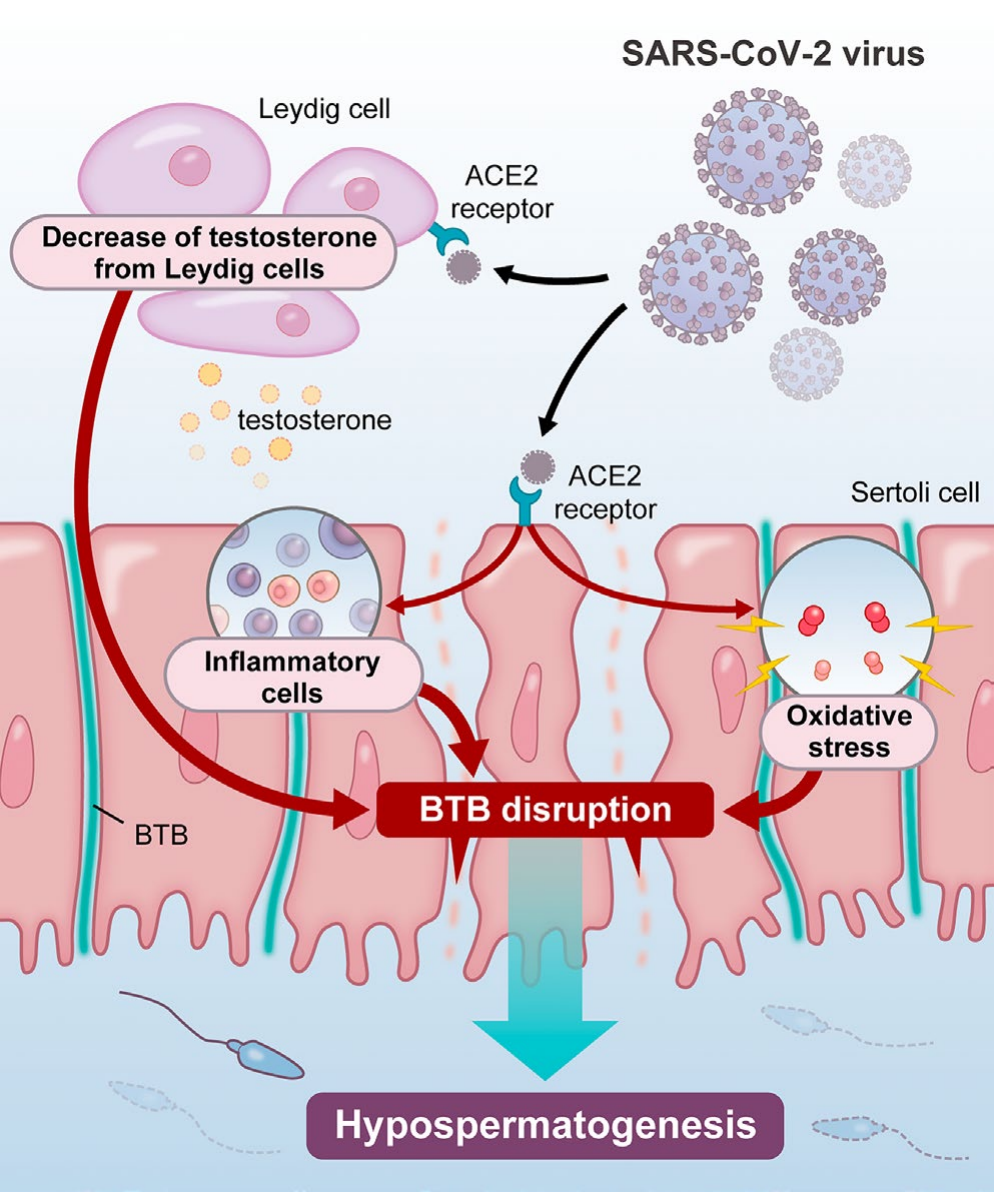
Schematic representation of hypothesized mechanisms of SARS‐CoV‐2‐induced impaired spermatogenesis SARS‐CoV‐2 primarily enters testicular cells through the angiotensin‐converting enzyme 2 (ACE2) receptor, which is highly expressed in Sertoli cells, Leydig cells, and spermatogonia. The binding of the virus to the ACE2 receptor facilitates its entry into these cells, resulting in direct viral‐induced cytopathic effects. Similar to the mumps virus, SARS‐CoV‐2 infection triggers a robust immune response. In severe cases, a cytokine storm characterized by elevated levels of IL‐6 and TNF‐α may occur. This systemic inflammation can extend to the testes, compromising the blood–testis barrier. In addition, Leydig cells, which are responsible for testosterone production, may be impaired. Increased oxidative stress is another potential mechanism. This is depicted by free radical symbols throughout the testicular tissue. These effects may collectively impair spermatogenesis and potential long‐term testicular function.

The COVID‐19 pandemic has posed unprecedented challenges to global health, including significant concerns regarding its effect on male reproductive health. SARS‐CoV‐2 can directly infect testicular cells, trigger systemic inflammation, and disrupt hormonal balance, which leads to impaired spermatogenesis (Figure 1). Identifying the underlying mechanisms for these effects, together with comprehensive monitoring and targeted therapeutic interventions, is essential to mitigate the long‐term consequences of COVID‐19 on male fertility.

Elevated LH levels and reduced testosterone ratios indicate potential Leydig cell dysfunction, with more severe disease correlating with greater hormonal disruption. These findings indicated the need for further studies to fully elucidate the mechanisms involved and to develop targeted interventions to mitigate the long‐term endocrine consequences of COVID‐19. Comprehensive monitoring of hormone levels and testicular function in COVID‐19 patients is essential for early detection and intervention.

Continued studies, vaccination efforts, and clinical vigilance are essential to address the reproductive health challenges posed by the ongoing COVID‐19 pandemic. Significant progress has been made in understanding the relationship between COVID‐19 infection and male infertility. Although data are limited, there is sufficient information to draw general conclusions and suggest future research directions.

Following infection, semen quality may be suppressed by mechanisms that reduce testosterone levels and affect various aspects of the semen profile, including sperm count, motility, morphology, and leukocyte infiltration. The extent of these effects depends on the severity and duration of the infection.

In summary, while reports suggest that spermatogenic dysfunction caused by COVID‐19 is generally mild and transient, the insights gained from studying the mechanisms of testicular dysfunction have broader implications. These findings may contribute to understanding and addressing spermatogenic dysfunction caused by emerging viral infections in the future. This review highlights the potential mechanisms involved, such as direct testicular infection, systemic inflammation, oxidative stress, and hormonal disruption, emphasizing their relevance for both current and future challenges in maintaining male reproductive health. Continued research and surveillance are essential to develop effective preventive and therapeutic strategies that ensure reproductive health in the face of evolving viral threats.

## CONFLICT OF INTEREST STATEMENT

Koji Chiba is an Editorial Board member of *Reproductive Medicine and Biology* and a co‐author of this article. To minimize bias, he was excluded from all editorial decision‐making related to the acceptance of this article for publication.
